# Platelet-to-Lymphocyte Ratio and Neutrophil-to-Lymphocyte Ratio in Patients With Newly Diagnosed Moyamoya Disease: A Cross-Sectional Study

**DOI:** 10.3389/fneur.2021.631454

**Published:** 2021-11-10

**Authors:** Wenyuan Ma, Changmeng Cui, Song Feng, Genhua Li, Guangkui Han, Jilan Liu, Xianyun Qin, Yawei Hu, Mengjie Wang, Lu Zhang, Feng Jin

**Affiliations:** ^1^Clinical Medical College, Jining Medical University, Jining, China; ^2^Shandong Provincial Key Laboratory of Stem Cells and Neuro-Oncology, Department of Neurosurgery, Affiliated Hospital of Jining Medical University, Jining, China

**Keywords:** moyamoya disease, inflammation, association, platelet-to-lymphocyte ratio, neutrophil-to-lymphocyte ratio

## Abstract

Inflammation has been proven to be one of the key factors in the pathogenesis of moyamoya disease (MMD). Platelet-to-lymphocyte ratio (PLR) and neutrophil-to-lymphocyte ratio (NLR) are cheap and reliable biomarkers of inflammation. Nevertheless, evidence regarding the relationship among PLR and NLR in patients with MMD is limited. The focus of this subject was to explore the relationship between PLR and NLR in patients with newly diagnosed MMD.

**Patients and methods:** A cross-sectional study was performed including 261 patients with diagnosed MMD for the first time who were enrolled from our hospital, from 24 March 2013 to 24 December 2018. The clinical characteristics were collected for each patient. Univariate analysis, smooth curve fitting and multivariate piecewise linear regression were showed.

**Results:** The mean levels or median values (interquartile range) of PLR and NLR were 146.979 ± 51.203 and 2.241 (1.589–2.984), respectively. A significant positive correlation between PLR and NLR levels (*P* < 0.001) was showed by the univariate analysis. Furthermore, a non-linear relationship was detected between PLR and NLR by smooth curve fitting after adjusting for potential confounders. A multivariate piecewise linear regression model revealed a significant positive correlation between PLR and NLR when the PLR level was lower than 219.82 (β 0.012, 95% CI 0.005, 0.019; *P* = 0.001). PLR was also significantly positively associated with NLR when PLR concentrations were >219.82 (β 0.098, 95% CI 0.069, 0.128; *P* < 0.001).

**Conclusion:** There seemed to be a positive association between PLR and NLR in patients with MMD. This may help to further explain the role of inflammation in the occurrence and progress of MMD.

## Introduction

Moyamoya disease (MMD) is a rare cerebrovascular disease characterized by progressive stenosis of large intracranial arteries and a hazy network of basal collaterals called moyamoya vessels (1). The disease may mainly lead to ischemic or hemorrhagic stroke. To date, the underlying mechanisms of MMD have remained to be fully demonstrated. With the deepening of research on MMD, systemic inflammation has been shown to play a critical role in the pathogenesis of MMD (2–4). A previous study reported a secure epidemiological association among MMD and certain diseases that have a segment of inflammation (5). These discoveries may demonstrate that in some patients with MMD, pathological vessel shapes may be a sequela of systemic inflammation (6). Currently, existing studies on MMD and inflammation have focused on inflammatory cytokines such as interleukin-1β (IL-1β), interleukin-12 (IL-12), transforming growth factor-β1 (TGF-β1) and tumor necrosis factor-α (TNF-α) (5, 7–9). Nevertheless, evidence regarding the relationship between platelet-to-lymphocyte ratio (PLR) and neutrophil-to-lymphocyte ratio (NLR) in patients with MMD is limited.

PLR and NLR are inexpensive and reproducible biomarkers of inflammation (10, 11). The relationship between PLR and NLR has been extensively studied in malignant tumors, systemic lupus erythematosus, rheumatoid arthritis and other diseases (12–15). PLR and NLR are reportedly associated with decreased overall survival or recurrence-free survival in patients with numerous cancers (16, 17). In addition, PLR and NLR have been identified as prognostic predictors of stroke (18). PLR is an integrated reflection of important opposite inflammatory pathways easily measured from a complete blood count. Platelet to lymphocyte ratio is a cheap tool and more predictive than either the platelet or the lymphocyte count alone (19). PLR originally served as a systemic inflammatory biomarker to predict the prediction of neoplastic diseases. The PLR was positively correlated with the standard of internal carotid artery stenosis (20). NLR serves as a secure prognostic index for patients suffering from various diseases. Neutrophil activation enhances the recruitment of a number of different cell types that are involved in acute and chronic inflammation (21). In addition, a high circulating NLR is a powerful biomarker of poor clinical event in numerous cancers (22). A previous study used 171 patients, which found that elevated NLR was independently associated with MMD (11).

The main purpose of this research is to clarify the relationship among PLR and NLR in patients with newly diagnosed MMD, which may help to further explain the role of inflammation in the occurrence and progress of MMD.

## Materials and Methods

### Patients Population

According to the hospital electronic medical record system, this cross-sectional study involved 261 patients with newly diagnosed MMD who visited to Department of Neurosurgery, Affiliated Hospital of Jining Medical University, Jining, Shandong, China, between 24 March 2013 and 24 December 2018.

The clinical code for each participant was performed according to the Guidelines for Diagnosis and Treatment of Moyamoya Disease (Spontaneous Occlusion of the Circle of Willis) (2012 Edition) (23). The diagnostic standard were as follows: 1. Cerebral angiography must show at least the following findings: (1) Stenosis or occlusion of the terminal portion of the intracranial internal carotid artery (ICA) or proximal portions of the anterior cerebral artery (ACA) and/or the middle cerebral artery (MCA), (2) Abnormal vascular networks in the vicinity of the occlusive or stenotic lesions in the arterial phase, and (3) Bilaterality of the findings in (1) and (2). 2. The following situations must have been eliminated: (1) atherosclerosis, (2) autoimmune disease, (3) meningitis, (4) brain tumors, (5) Down's syndrome, (6) von Recklinghausen's disease, (7) head injury, (8) cerebrovascular lesions after head irradiation, and (9) others.

The selection criteria included patients hospitalized in our hospital who were diagnosed with MMD for the first time. The elimination criteria were: (1) patients with hematological diseases, malignancy, autoimmune diseases, metabolic diseases, or existing infections; (2) patients treated with glucocorticoid, permanent immunomodulatory drugs or anti-inflammatory drugs; (3) patients younger than 18 years old and (4) patients who have undergone MMD operation (24, 25).

The study was authorized by the Ethics Committee of Affiliated Hospital of Jining Medical University (protocol number 2020C034) and informed approve was abandoned due to the retrospective study design.

### Data Collection

All patients' demographic and clinical information on admission were retrospectively gathered, including age; sex; body mass index (BMI); smoking status; alcohol consumption; diabetes; hypertension; blood routine index; type of onset; Suzuki stage. When patients were admitted to the hospital, obtained the blood routine index using the method in our previous study (26). PLR was calculated as platelet counts divided by those of lymphocyte and NLR was calculated as neutrophil counts divided by lymphocyte counts using the same blood samples drawn.

### Statistical Analysis

We show continuous variables with a normal distribution as the mean ± standard deviation, and we gave continuous variables with skewed distributions as the medians (Q1–Q3). Categorical variables were gave as frequencies or percentages. First, we describe the demographic characteristics and blood routine index of the subjects. Next, a univariate analysis pattern was used to measure the significance of the association between PLR and NLR as well as the other separated variables. Then, to explore the non-linearity of PLR and NLR, we implemented a generalized additive model and smooth curve fitting. If non-linearity was identified, we first calculated the inflection point using the recursive algorithm and then created a two-piecewise linear regression on both sides of the inflection point. Finally, we divided MMD patients into patients with intracranial ischemia and intracranial hemorrhage according to the type of onset and investigated the relationship among PLR and NLR by smooth curve fitting after adjustment for possible confounders. All analyses were gave with the R statistical software packages (http://www.R-project.org, The R Foundation) and EmpowerStats (http://www.empowerstats.com, X&Y Solutions, Inc, Boston, MA). *P*-values < 0.05 (two-sided) were considered statistically significant.

## Results

### Baseline Characteristics of the Selected Participants

The clinical characteristics of all the patients were described in Table 1. A total of 252 patients, consisting of 123 (48.8%) males and 129 (51.2%) females, were selected. The average age of the participants was 48.9 ± 10.2 years. The participants included 195 (77.4%) cases of intracranial ischemia, 56 (22.2%) cases of intracranial hemorrhage and 1 (0.4%) case of seizure. One (0.4%) patient was in Suzuki stage 1, 15 (5.9%) patients were in Suzuki stage 2, 160 (63.5%) patients were in Suzuki stage 3, 66 (26.2%) patients were in Suzuki stage 4, and 10 (4%) patients were in Suzuki stage 5. The average levels or median values (interquartile range) of PLR and NLR were 146.979 ± 51.203 and 2.241 (1.589–2.984), respectively.

**Table 1:** Baseline characteristics of the participants.

**Variables**	**All**
Number	252
Age (years, mean ± sd)	48.9 ± 10.2
BMI (kg/m^2^, mean ± sd)	25.57 ± 3.35
Platelet (10^9^/L, mean ± sd)	244.673 ± 61.025
Neutrophil (10^9^/L, median, Q1–Q3)	3.890 (2.910–5.000)
Lymphocyte (10^9^/L, mean ± sd)	1.759 ± 0.577
NLR (median, Q1–Q3)	2.241 (1.589–2.984)
PLR (mean ± sd)	146.979 ± 51.203
Sex, *n* (%)	
Female	129 (51.2%)
Male	123 (48.8%)
Smoking status, *n* (%)	
No	178 (71.2%)
Yes	72 (28.8%)
Alcohol consumption, *n* (%)	
No	184 (73.6%)
Yes	66 (26.4%)
Diabetes, *n* (%)	
No	235 (93.3%)
Yes	17 (6.7%)
Hypertension, *n* (%)	
No	171 (67.9%)
Yes	81 (32.1%)
Type of onset, *n* (%)	
Intracranial ischemia	195(77.4%)
Intracranial hemorrhage	56 (22.2%)
Seizure	1 (0.4%)
Suzuki stage, *n* (%)	
Stage 1	1 (0.4%)
Stage 2	15 (5.9%)
Stage 3	160 (63.5%)
Stage 4	66 (26.2%)
Stage 5	10 (4%)

*BMI, body mass index; PLR, platelet-to-lymphocyte ratio; NLR, neutrophil-to-lymphocyte ratio*.

### Univariate Analysis for NLR

Univariate linear regression test was performed to measure the relationships between all tested variables and NLR. As shown in Table 2, for the unadjusted pattern, a significant positive relationship between NLR and PLR was observed (*P* < 0.001). We also observed a significant positive correlation between NLR and neutrophil (*P* < 0.001) and lymphocyte (*P* < 0.001) were significant negatively associated with NLR. No association was observed between NLR and sex, age, BMI, smoking status, alcohol consumption, diabetes, hypertension or platelet (*P* > 0.05).

**Table 2:** Univariate analysis for NLR.

**Covariate**	**Statistics**	***β*** **(95% CI)**	***P-*** **value**
Sex			
Female	129 (51.2%)	Reference	
Male	123 (48.8%)	0.474 (−0.398, 1.346)	0.288
Age, years	48.9 ± 10.2	0.004 (−0.038, 0.047)	0.840
BMI, kg/m^2^	25.57 ± 3.35	0.018 (−0.133, 0.170)	0.811
Smoking status			
No	178 (71.2%)	Reference	
Yes	72 (28.8%)	0.432 (−0.531, 1.395)	0.381
Alcohol consumption			
No	184 (73.6%)	Reference	
Yes	66 (26.4%)	0.902 (−0.082, 1.886)	0.074
Diabetes, *n* (%)			
No	235 (93.2%)	Reference	
Yes	17 (6.7%)	0.570 (−1.149, 2.288)	0.517
Hypertension, *n* (%)			
No	171 (67.9%)	Reference	
Yes	81 (32.1%)	−0.119 (−1.051, 0.813)	0.803
Platelet	244.673 ± 61.025	−0.001 (−0.008, 0.006)	0.773
Neutrophil	4.507 ± 2.703	1.033 (0.936, 1.130)	<0.001
Lymphocyte	1.759 ± 0.577	−2.845 (−3.515, −2.175)	<0.001
PLR	146.979 ± 51.203	0.023 (0.017, 0.028)	<0.001

*BMI, body mass index; PLR, platelet-to-lymphocyte ratio*.

### Independent Correlation Between PLR and NLR by Multivariate Piecewise Linear Regression

**Figure 1:**
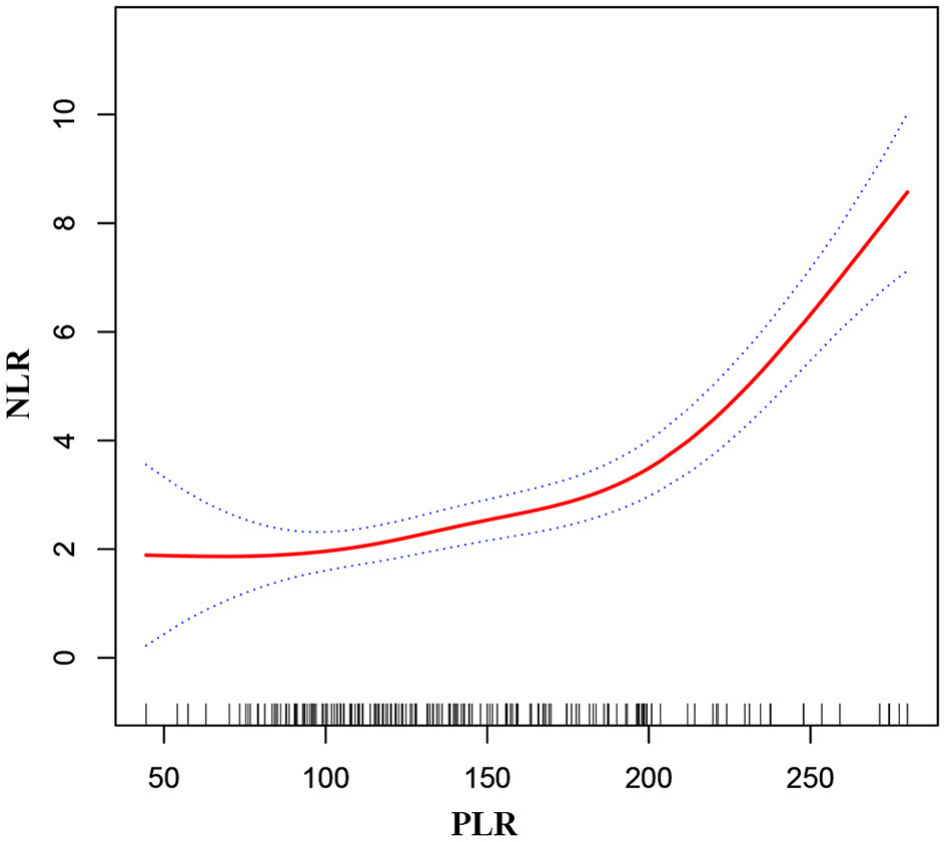
Association between PLR and NLR in MMD patients. The solid red line stands for the smooth curve fit between the variables. Blue bands stand for the 95% confidence interval of the fit. The pattern was adjusted for sex, age, BMI, smoking status, and alcohol consumption.

As shown in Figure 1, smooth curve fitting was performed after adjusting for possible confounding factors, including sex, age, BMI, smoking status and alcohol consumption. The participants' NLR levels demonstrated an non-linear relationships with PLR. Specifically, the NLR levels displayed an increasing trend as the PLR increased. As shown in Table 3, we further analyzed the threshold effect based on curve fitting. We observed a significant positive correlation between PLR and NLR when the PLR level was lower than 219.82 (β 0.012, 95% CI 0.005, 0.019; *P* = 0.001). PLR was also significantly positively associated with NLR when PLR concentrations were >219.82 (β 0.098, 95% CI 0.069, 0.128; *P* < 0.001).

**Table 3:** The result of two-piecewise linear regression model.

**Inflection point of PLR**	**Effect size *(β)***	**95% CI**	***P-*** **value**
<219.82	0.012	0.005, 0.019	0.001
≥219.82	0.098	0.069, 0.128	<0.001

*PLR, platelet-to-lymphocyte ratio*.

### Independent Correlation Between PLR and NLR by Multivariate Piecewise Linear Regression in MMD Patients With Intracranial Ischemia or Intracranial Hemorrhage

**Figure 2:**
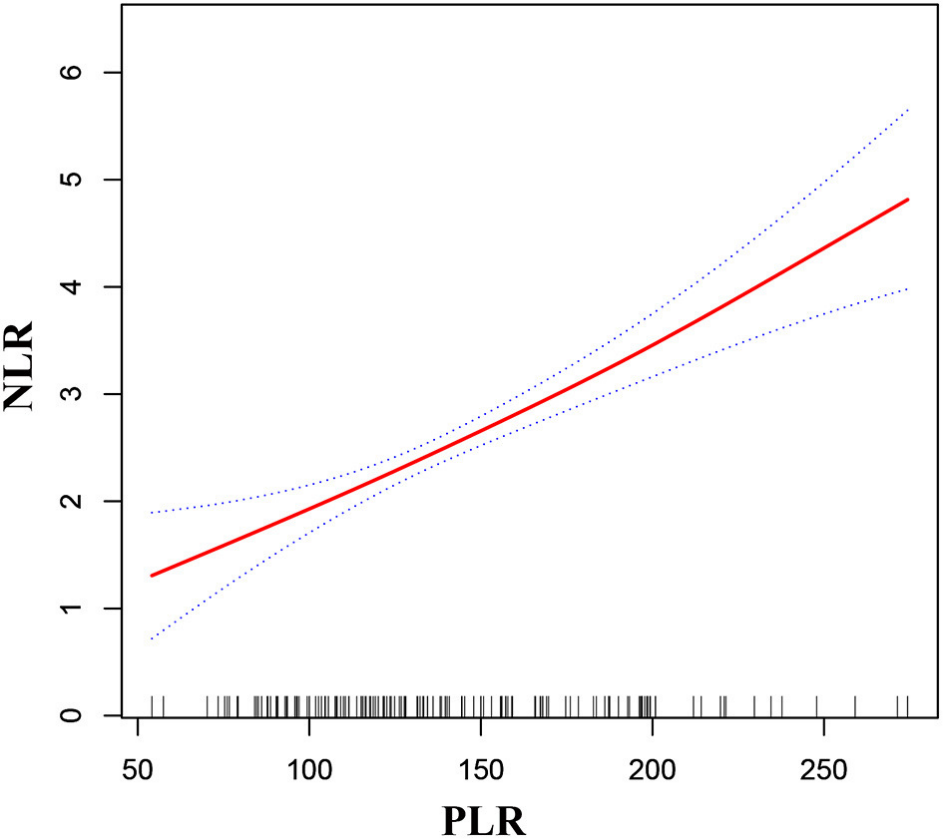
Association between PLR and NLR in MMD patients with intracranial ischemia. The solid red line stands for the smooth curve fit between the variables. Blue bands stand for the 95% confidence interval of the fit. The pattern was adjusted for sex, age, BMI, smoking status, and alcohol consumption.

**Figure 3:**
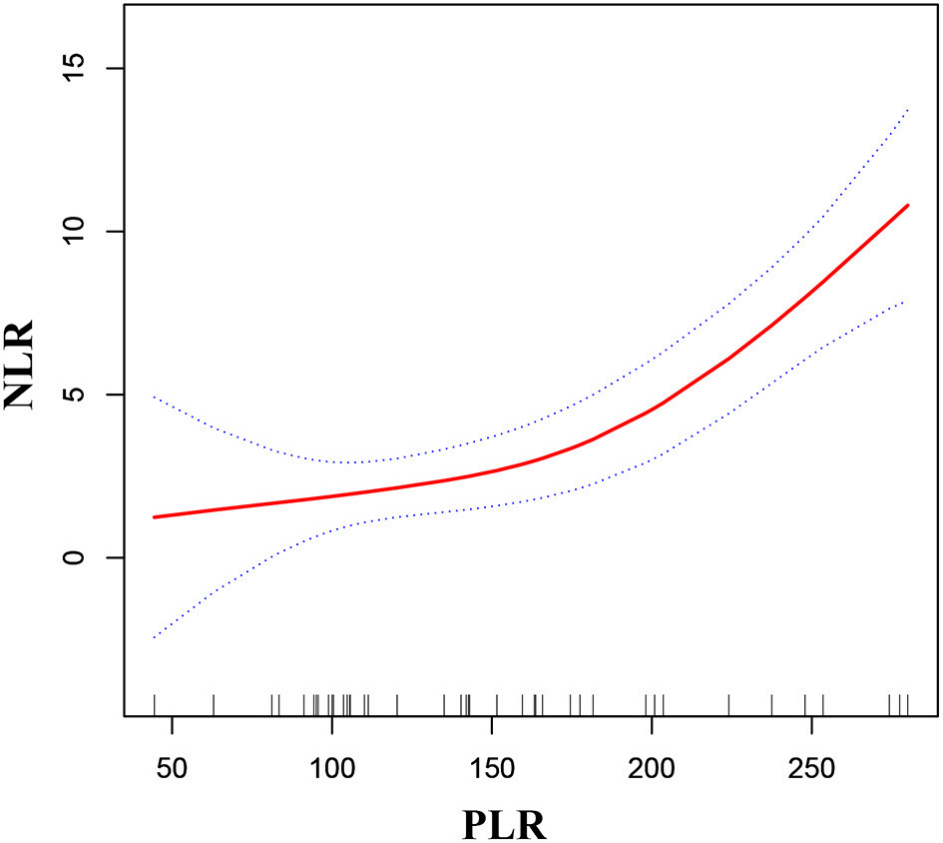
Association between PLR and NLR in MMD patients with intracranial hemorrhage. The solid red line stands for the smooth curve fit between the variables. Blue bands stand for the 95% confidence interval of the fit. The pattern was adjusted for sex, age, BMI, smoking status, and alcohol consumption.

As shown in Figures 2, 3, smooth curve fitting was showed in MMD patients with intracranial ischemia or intracranial hemorrhage according to the method in Figure 1. We found that NLR levels displayed an increasing trend as PLR increased in MMD patients with intracranial ischemia and intracranial hemorrhage.

## Discussion

MMD was defined in 1969 by Suzuki and Takaku (27) as a distinct cerebrovascular disease that is characterized by progressive stenosis or occlusion of the bilateral internal carotid arteries with unknown etiology, and plentiful collateral vessels appear at the base of the brain (28). The disease generally occurs in East Asian populations, including pediatric and adult patients, and may bring about ischemic or hemorrhagic stroke, headaches, epilepsy, or transient ischemic attack (29). For a long time, researchers have carried out in-depth research on the underlying mechanisms of MMD. Up to now, the mechanisms through which MMD occurs and evolves remain unidentified. Some researches manifest that MMD could be the result of vascular immune injury and inflammation response (2, 30). Recent studies of our team have found that the upregulated expressions of various plasma inflammatory factors, such as IL-1β, IL-12, and TNF-α, in patients with MMD suggested that inflammation might regulate the process of MMD (31). Inflammatory response ultimately leads to the hyperplasia of intimal vascular smooth muscle cells and neovascularization by proliferation of endothelial cells, that motivates lumen stenosis and collateral formation (32).

PLR and NLR are reliable marker of systemic inflammation. Although there have been extensive investigations on NLR and PLR, the normal ranges of NLR and PLR were less investigated. It was reported that the average NLR is 2.15 in the United States population (33) and 1.65 in South Korea (34), which suggested that NLR is race specific. A study investigated the reference range of NLR and PLR in Chinese Han population from Chaoshan region in South China. Researchers found that the 95% reference range of NLR in normal male and female are 0.43–2.75 and 0.37–2.87, PLR are 36.63–149.13 and 43.36–172.68, respectively (35).

The PLR and NLR have been regarded indicators of systemic inflammation in numerous present clinical studies, when patients have no evident infection (36). Therefore, the evaluation of such cheap and readily available prognostic indicator was essentially needed for large numbers of experiments. It is well-known that, while the number of neutrophils and platelets increase, lymphocytes decrease during an inflammation. As neutrophil and platelet counts increase, they secrete numerous kinds of inflammatory factors, which mean a stronger inflammatory response (12). Platelets play an dynamic role in inflammation. One of the most important mechanisms of platelets was their capacity to collect leukocytes to sites of inflammation. Platelets have capacity to create aggregates with neutrophils and monocytes, and also motivate an inflammatory monocyte phenotype (37). In addition, autopsy studies have demonstrated smooth muscle proliferation, infiltrating macrophages, and T lymphocyte in moyamoya vessel walls (6). The PLR and NLR can exhibit the inflammatory state and predict the prediction of the tumor (38). Recently, it has been reported that PLR and NLR can be managed to independently predict 90-day practical outcome in patients after primary brainstem hemorrhage (39). PLR has been shown as a novel indicator for major adverse outcomes in cardiovascular disorders (37). In patients with mitral annular calcification, there was a positive correlation between the PLR and NLR (40). Aortic stenosis was a progressive disease related with inflammation. PLR had significant positive correlation with NLR in patients with aortic stenosis (37). In our study, a non-linear positive relationship was observed between PLR and NLR in patients with newly diagnosed MMD. When we divided MMD patients into patients with intracranial ischemia and intracranial hemorrhage according to the type of onset, we found that NLR levels displayed an increasing trend as PLR increased in MMD patients with intracranial ischemia and intracranial hemorrhage. The clinical value of this study is as follows. (1) To the best of our knowledge, this is the first study to detect the independent association between PLR and NLR in patients with newly diagnosed MMD. (2) The relationship between PLR and NLR may help to further explain the role of inflammation in the occurrence and development of MMD. (3) Our study could enrich the role of PLR and NLR as inflammatory markers in various diseases.

There are some limitations in the current study. First, in this study, our consider subjects were patients with newly diagnosed MMD. Therefore, there is a definite deficiency in the universality and extrapolation of the study. Second, the dynamic changes of NLR and PLR are not explored because of unavoidable selection bias and assessment bias. Thirdly, because we excluded patients with hematological diseases, malignancy, autoimmune diseases, metabolic diseases or existing infections, patients treated with glucocorticoid, permanent immunomodulatory drugs or anti-inflammatory drugs and patients younger than 18 years old, the discoveries of this research cannot be generalized to these people. Lastly, the hospitalized patients with MMD did not used the modified Rankin Scale at the time. Only some patients have tested the cytokines. We will add these two parts in our follow-up research.

## Conclusion

In conclusion, the present study showed a positive association between PLR and NLR among patients with newly diagnosed MMD. This may help to further explain the role of inflammation in the occurrence and progress of MMD.

## Data Availability Statement

The raw data supporting the conclusions of this article will be made available by the authors, without undue reservation.

## Ethics Statement

The study was approved by the Ethics Committee of Affiliated Hospital of Jining Medical University (protocol number 2020C034) and informed consent was waived due to the retrospective study design.

## Author Contributions

WM, SF, GL, and GH were involved in the study design. JL, XQ, YH, MW, and LZ were responsible for the data collection. WM analyzed data and wrote the manuscript. FJ and CC modified and revised the manuscript. All authors have read and approved the final version of the manuscript.

## Funding

This work was supported by the Key Research and Development Program of Jining Science and Technology (2018SMNS005), a Project of Shandong Province Medical Health and Technology Development Program (2014WS0518), and Research Support Fund for Teachers of Jining Medical University (JYFC2018FKJ100).

## Conflict of Interest

The authors declare that the research was conducted in the absence of any commercial or financial relationships that could be construed as a potential conflict of interest.

## Publisher's Note

All claims expressed in this article are solely those of the authors and do not necessarily represent those of their affiliated organizations, or those of the publisher, the editors and the reviewers. Any product that may be evaluated in this article, or claim that may be made by its manufacturer, is not guaranteed or endorsed by the publisher.
